# Supplementary figures and images for: Testing biological actions of medicinal plants from northern Vietnam on zebrafish embryos and larvae: Developmental, behavioral, and putative therapeutical effects
Source: PLoS One. 2023 Nov 7;18(11):e0294048. doi: 10.1371/journal.pone.0294048 (PMC10629648; doi:10.1371/journal.pone.0294048)

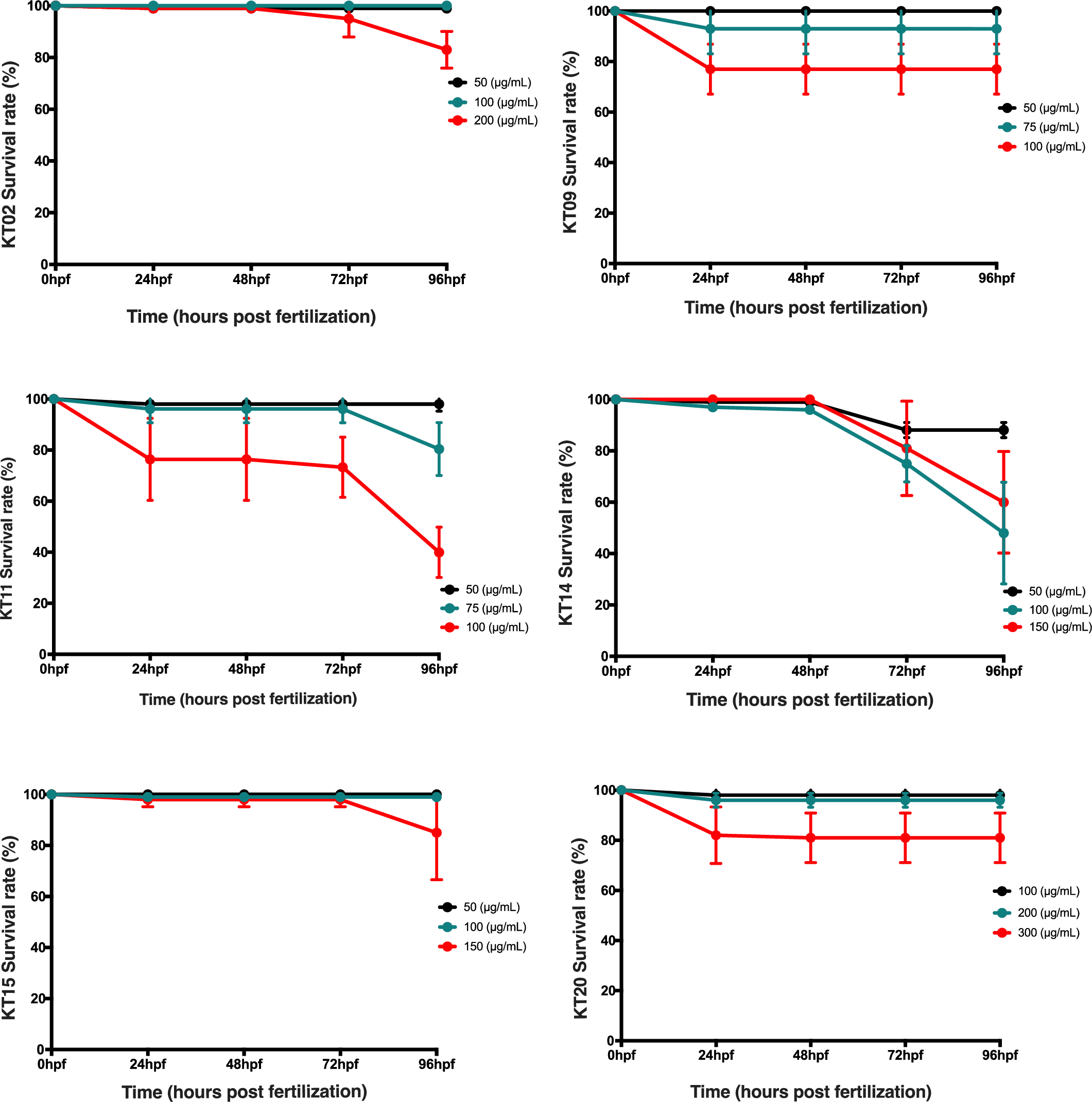

Supplement: S1 Fig — (TIF) [file pone.0294048.s001.tif]

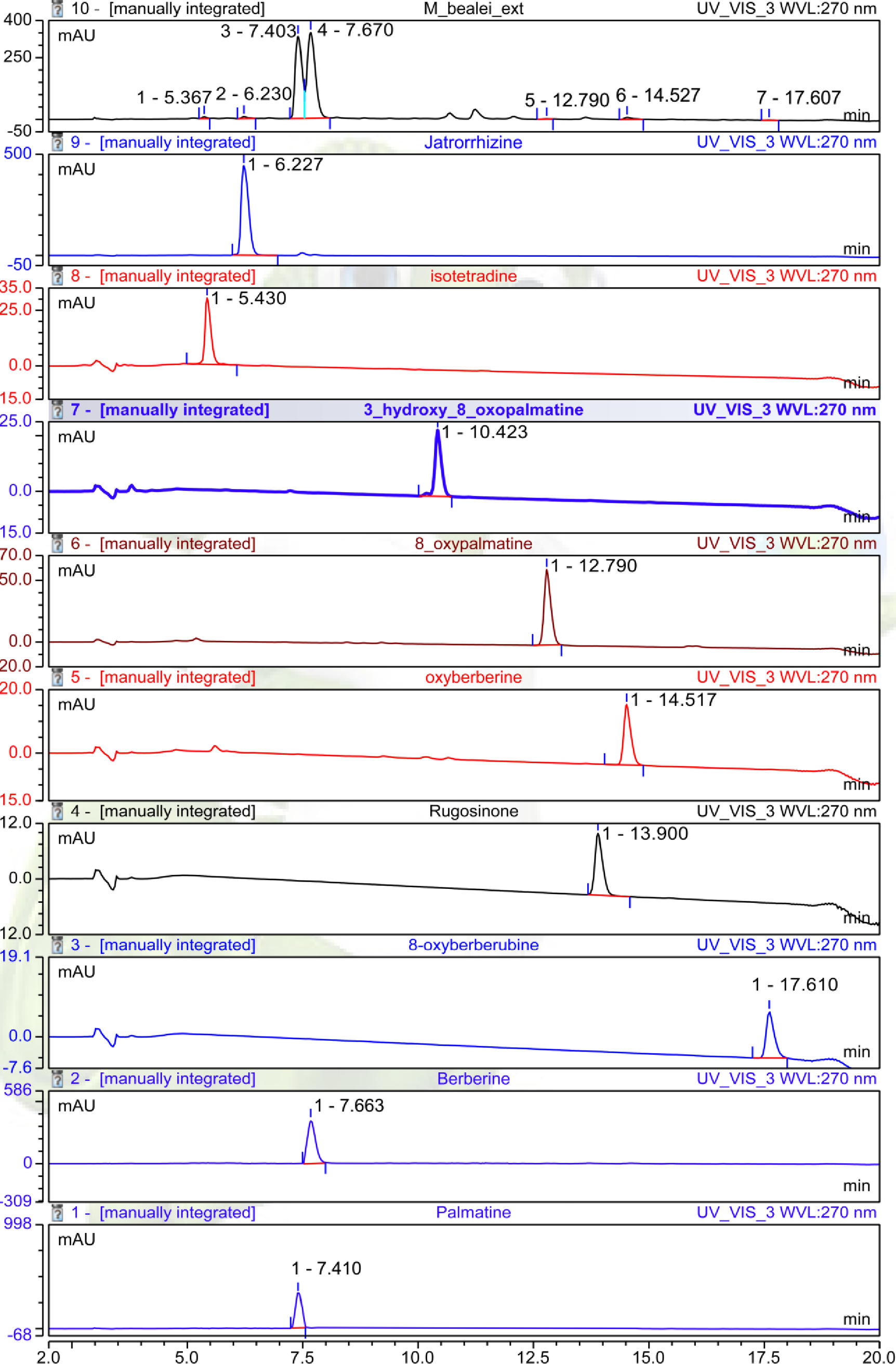

Supplement: S2 Fig — HPLC chromatograms of M. bealei extract and in-house reference compounds, including jatrorrhizine, isotetradine, 3-hydroxy-8-oxopalmatine, 8-oxypalmatine, oxyberberine, rugosinone, 8-oxyberberrubine, berberine, and palmatine. (TIF) [file pone.0294048.s002.tif]

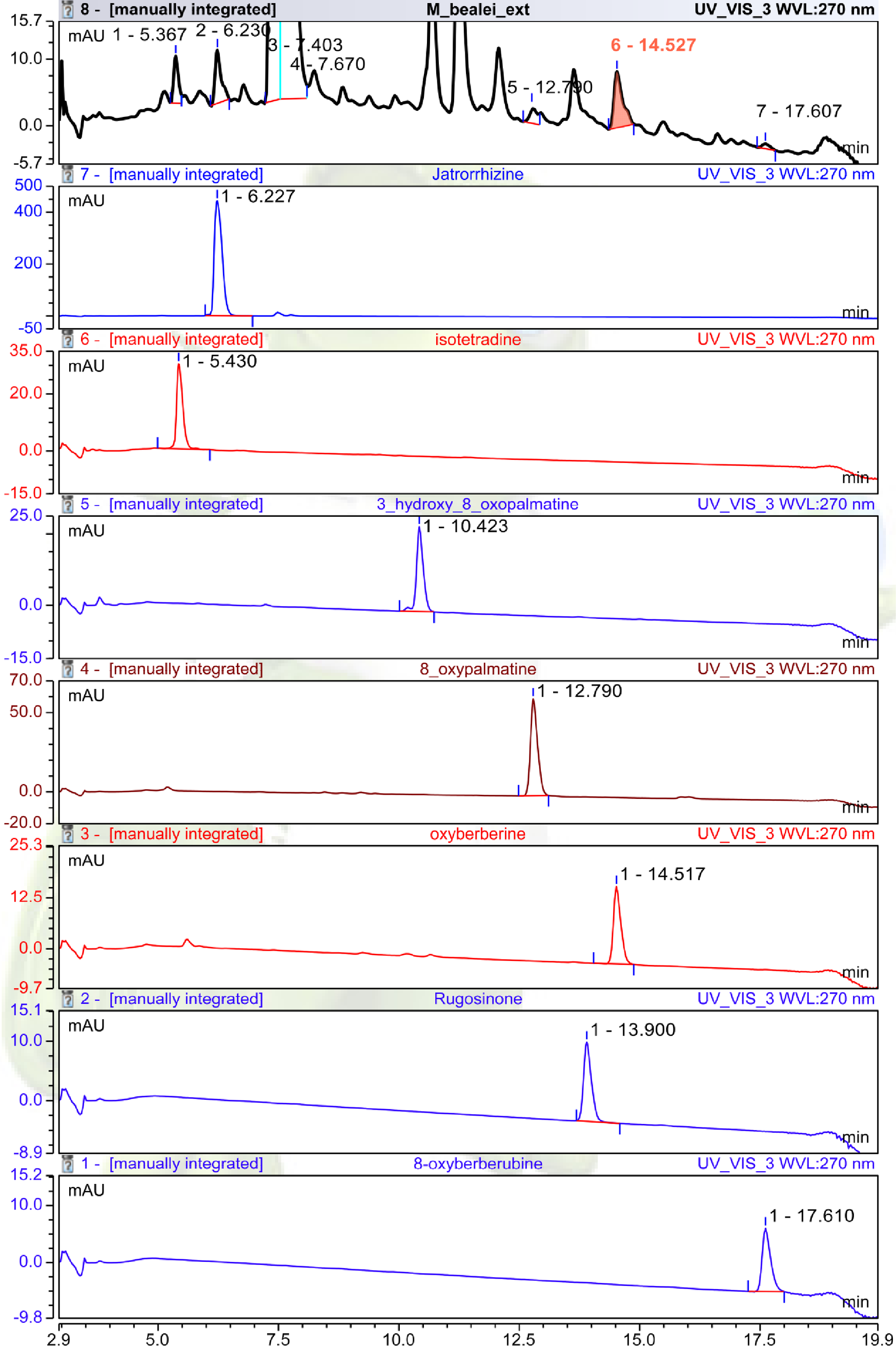

Supplement: S3 Fig — Extended chromatograms (-6 to 16 mAu) of M. bealei extract, jatrorrhizine, isotetradine, 3-hydroxy-8-oxopalmatine, 8-oxypalmatine, oxyberberine, rugosinone, and 8-oxyberberrubine. Matched compounds (jatrorrhizine, isotetradine, 8-oxypalmatine, oxyberberine, and 8-oxyberberrubine) are marked on the chromatogram of the extract. (TIF) [file pone.0294048.s003.tif]

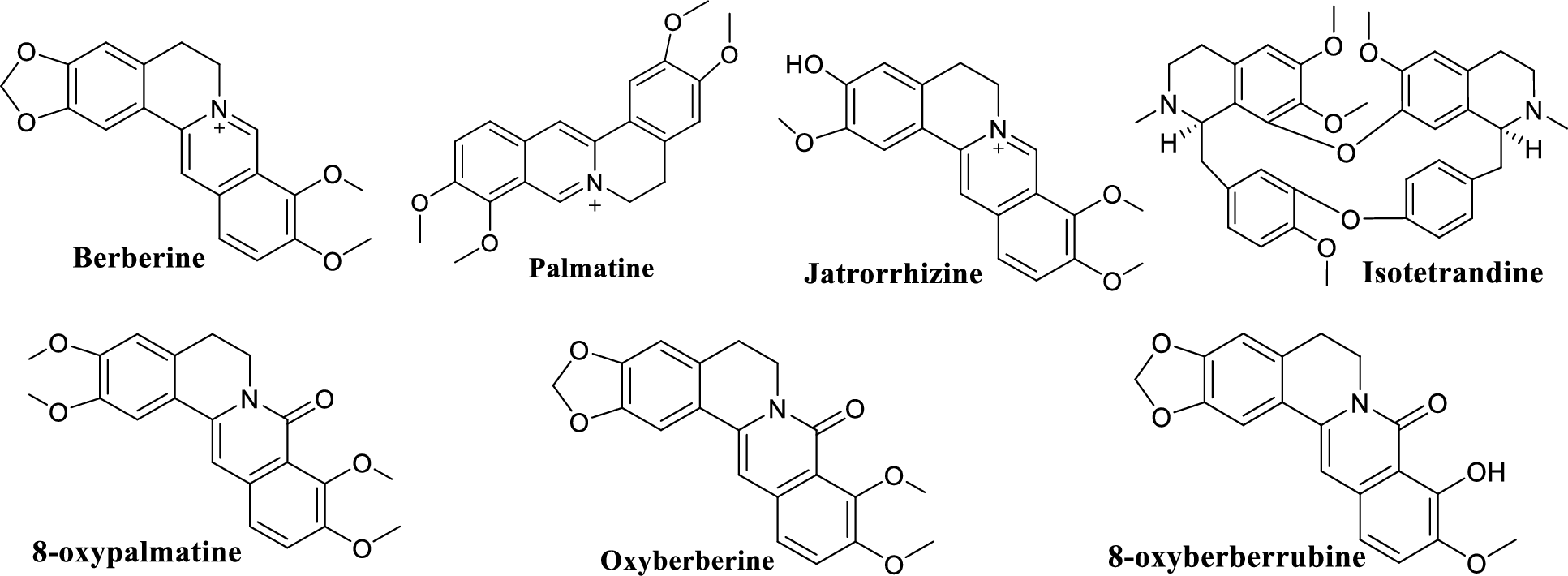

Supplement: S4 Fig — (TIF) [file pone.0294048.s004.tif]
