# Supplementary material for: Testing biological actions of medicinal plants from northern Vietnam on zebrafish embryos and larvae: Developmental, behavioral, and putative therapeutical effects
Source: PLoS One. 2023 Nov 7;18(11):e0294048. doi: 10.1371/journal.pone.0294048 (PMC10629648; doi:10.1371/journal.pone.0294048)
Supplement: S2 Table — (DOCX) [file pone.0294048.s006.docx]

**Table S2. Genes most affected by KY09 treatment**

| **Gene** | **ID** | **log2(Fold-Change)** | **p-adj** |
| --- | --- | --- | --- |
| morc3b | ENSDARG00000043271 | 3.38 | 2.15E-28 |
| myo1g | ENSDARG00000036104 | 3.27 | 1.27E-20 |
| adgrg11 | ENSDARG00000041413 | 3.25 | 6.80E-16 |
| arhgap45a | ENSDARG00000052496 | 2.73 | 2.79E-41 |
| bsnb | ENSDARG00000079161 | 2.59 | 1.06E-16 |
| dachb | ENSDARG00000034785 | 2.56 | 4.23E-23 |
| socs4 | ENSDARG00000078509 | 2.49 | 8.93E-21 |
| npdc1b | ENSDARG00000056156 | 2.45 | 2.83E-12 |
| egln3 | ENSDARG00000032553 | 2.41 | 2.48E-37 |
| pycr1b | ENSDARG00000098639 | 2.27 | 8.50E-12 |
| cxcr4b | ENSDARG00000041959 | 2.25 | 3.57E-24 |
| adap2 | ENSDARG00000070565 | 2.23 | 2.59E-19 |
| sebox | ENSDARG00000042526 | 2.19 | 5.94E-16 |
| mfsd4aa | ENSDARG00000023768 | 2.18 | 3.93E-16 |
| plxdc2 | ENSDARG00000059950 | -2.77 | 2.89E-79 |
| pip5k1ca | ENSDARG00000076001 | -2.78 | 1.90E-12 |
| hoxc8a | ENSDARG00000070346 | -2.78 | 1.49E-14 |
| nfasca | ENSDARG00000061099 | -2.79 | 1.64E-21 |
| hoxb10a | ENSDARG00000011579 | -2.84 | 2.91E-09 |
| pax8 | ENSDARG00000015879 | -2.86 | 3.70E-19 |
| kctd15a | ENSDARG00000045893 | -2.90 | 7.55E-18 |
| grin2db | ENSDARG00000070620 | -2.97 | 1.55E-19 |
| col4a6 | ENSDARG00000052061 | -2.99 | 7.70E-36 |
| gabrp | ENSDARG00000020901 | -3.00 | 7.92E-27 |
| arhgap21a | ENSDARG00000104295 | -3.04 | 2.40E-23 |
| hoxb8a | ENSDARG00000056027 | -3.10 | 2.60E-16 |
| cdh11 | ENSDARG00000021442 | -3.10 | 5.12E-33 |
| pax6b | ENSDARG00000045936 | -3.23 | 2.22E-47 |
| fezf2 | ENSDARG00000070677 | -3.26 | 5.48E-22 |
| ccndx | ENSDARG00000019741 | -3.40 | 1.51E-22 |
| hoxb3a | ENSDARG00000029263 | -3.42 | 3.64E-34 |
| pax3a | ENSDARG00000010192 | -3.51 | 3.71E-22 |
| fhdc1 | ENSDARG00000074812 | -3.60 | 2.67E-23 |
| nr2f5 | ENSDARG00000033172 | -3.86 | 1.47E-24 |
| wnt8b | ENSDARG00000006911 | -4.04 | 2.24E-26 |
| hoxb7a | ENSDARG00000056030 | -4.14 | 3.11E-26 |
| hoxb9a | ENSDARG00000056023 | -4.44 | 4.89E-35 |
| hoxc3a | ENSDARG00000070339 | -4.70 | 4.97E-25 |
