# Supplementary material for: Testing biological actions of medicinal plants from northern Vietnam on zebrafish embryos and larvae: Developmental, behavioral, and putative therapeutical effects
Source: PLoS One. 2023 Nov 7;18(11):e0294048. doi: 10.1371/journal.pone.0294048 (PMC10629648; doi:10.1371/journal.pone.0294048)
Supplement: S3 Table — (DOCX) [file pone.0294048.s007.docx]

**Table S4. GSEA – GO term analysis**

|  | **Gene Set** | **Description** | **FDR** |
| --- | --- | --- | --- |
| **GO-BP** | GO :0048880 | sensory system development | 0.00E+00 |
|  | GO :0060322 | head development | 0.00E+00 |
|  | GO :0009792 | embryo development ending in birth or egg hatching | 0.00E+00 |
|  | GO :0007423 | sensory organ development | 0.00E+00 |
|  | GO :0007417 | central nervous system development | 0.00E+00 |
|  | GO :0009891 | positive regulation of biosynthetic process | 4.97E-04 |
|  | GO :0030182 | neuron differentiation | 8.52E-04 |
|  | GO :0007389 | pattern specification process | 8.70E-04 |
|  | GO :0045935 | positive regulation of nucleobase-containing compound metabolic process | 8.84E-04 |
|  | GO :2000026 | regulation of multicellular organismal development | 1.27E-03 |
|  | GO :0010628 | positive regulation of gene expression | 1.29E-03 |
|  | GO :0045595 | regulation of cell differentiation | 1.45E-03 |
|  | GO :0048598 | embryonic morphogenesis | 1.57E-03 |
|  | GO :0045165 | cell fate commitment | 1.59E-03 |
|  | GO :0060485 | mesenchyme development | 1.70E-03 |
|  | GO :0019932 | second-messenger-mediated signaling | 1.16E-02 |
|  | GO :0000003 | reproduction | 1.61E-02 |
|  |  |  |  |
| **GO-MF** | GO :0008134 | transcription factor binding | 0.00E+00 |
|  | GO :0000981 | DNA-binding transcription factor activity, RNA polymerase II-specific | 0.00E+00 |
|  | GO :0003690 | double-stranded DNA binding | 0.00E+00 |
|  | GO :0001067 | regulatory region nucleic acid binding | 0.00E+00 |
|  | GO :0046983 | protein dimerization activity | 3.16E-04 |
|  | GO :0030545 | receptor regulator activity | 9.48E-02 |
|  | GO :0003712 | transcription coregulator activity | 1.19E-01 |
